# Assessing the influence of gastrocnemius reconstruction on stress distribution of femoral tumor rotating hinge knee prosthesis via finite element analysis

**DOI:** 10.3389/fbioe.2024.1391298

**Published:** 2024-04-19

**Authors:** Jie Jiang, Fanwei Zeng, Xiaodong Tang

**Affiliations:** ^1^ Department of Musculoskeletal Tumor, People’s Hospital, Peking University, Beijing, China; ^2^ Beijing Key Laboratory of Musculoskeletal Tumor, Beijing, China

**Keywords:** bone tumor, rotating hinge knee prosthesis, gastrocnemius reconstruction, anterior tibial translation, finite element analysis

## Abstract

**Background:**

After femoral oncological knee arthroplasty, some patients suffer from rotating axis fracture, which significantly impacts the life span of the rotating hinge knee (RHK) prosthesis. This research aimed to analyze the biomechanical response of anatomical gastrocnemius reconstruction and assess whether it could reduce the risk of rotating axis breakage by finite element (FE) analysis.

**Methods:**

A femur-prosthesis-tibia FE model was established using the data from CT scans. The mechanical properties of the RHK implant were quantitatively compared before and after gastrocnemius reconstruction at 6 angles: 10°, 20°, 30°, 40°, 50°, and 60°.

**Results:**

Our results showed that gastrocnemius reconstruction effectively altered the stress distribution around the rotating axis, considerably relieving the stress in the fracture-prone region. In addition, the peak stress in the rotating axis, bending axis, prosthesis stem, and femoral condyles decreased variably.

**Conclusion:**

In distal femoral resection knee arthroplasty, the rebuilding of gastrocnemius substantially improved the stress distribution within the prosthesis, thereby having the potential to reduce the risk of prosthetic fracture and prolong the overall durability of the prosthesis.

## Introduction

**FIGURE 1:**
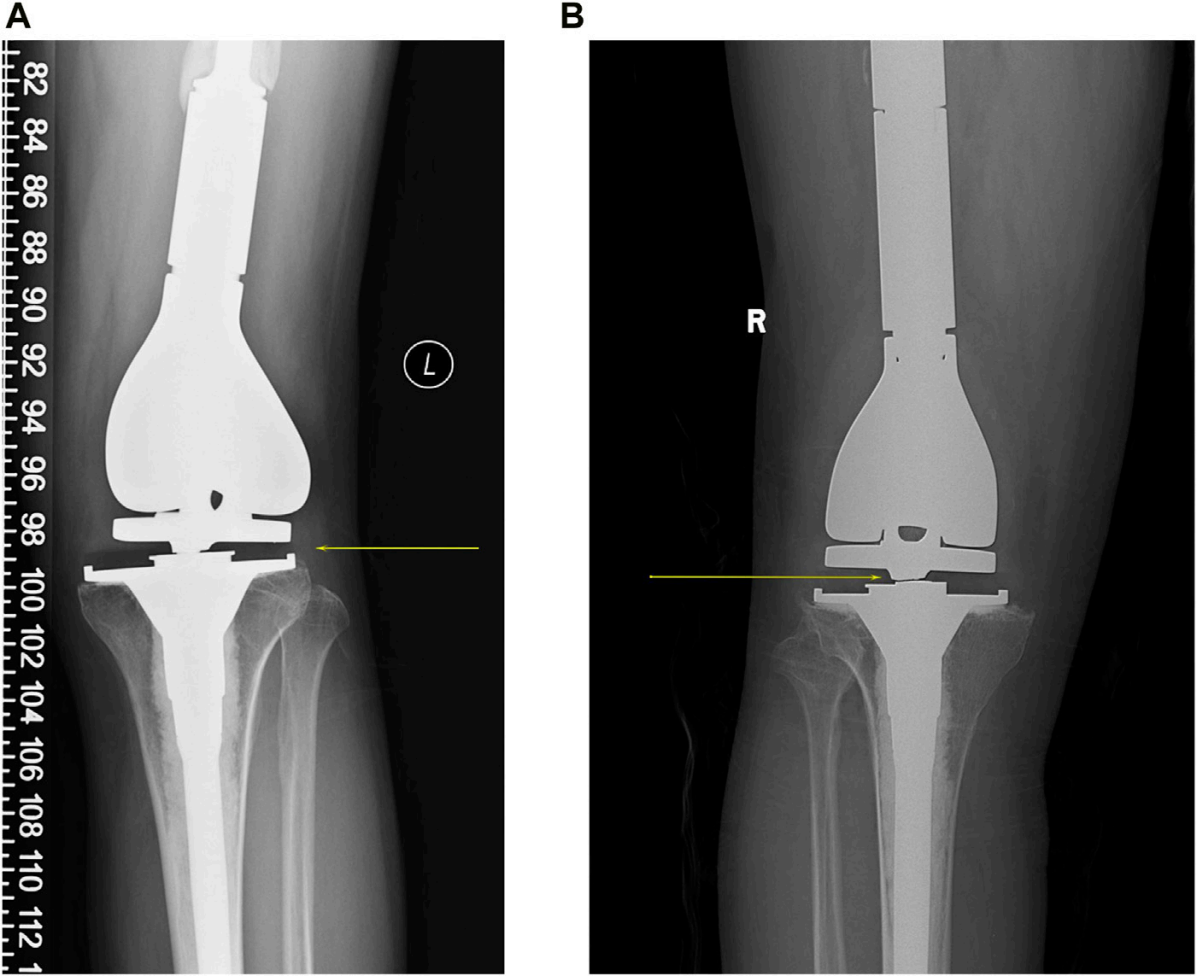
Two cases of rotating axis fractures of the tumor knee prosthesis, with yellow arrows showing the fracture sites. **(A)** A 36-year-old man had a prosthesis inserted for an undifferentiated sarcoma of the distal left femur. Subsequent to 5 years, the rotating axis fractured, requiring a comprehensive knee revision. **(B)** A 42-year-old man underwent the replacement of an artificial prosthesis due to the giant cell tumor of bone in his right knee. After 6 years, a rotating axis fracture within the prosthesis led him to a revision of the knee prosthesis.

Malignant bone tumors frequently occur in the knee joint, especially the distal femur (Ritter and Bielack, 2010). Over the past 30 years, with advances in medical imaging technology, chemotherapy, and surgery, limb salvage has replaced amputation as the standard treatment for malignant bone tumors (Grimer, 2005; Bielack et al., 2009; Beird et al., 2022). Currently, oncoplastic joint prostheses are widely used to reconstruct large bone defects after tumor resection. They provide immediate postoperative stability, early weight-bearing ambulation, and better long-term function (Plötz et al., 2002; Frink et al., 2005). However, endoprosthetic failures remain a significant concern, consisting of structural failure, soft-tissue failure, aseptic loosening, infection, and tumor progression (Henderson et al., 2011; Haijie et al., 2018). In some cases, we have noted prosthetic rotating axis fractures, which occur primarily in the spacer-wrapped area of the axis, greatly affecting the longevity of the prosthetic implants (Figure 1). This may be related to anterior shear forces exerted on the proximal tibia by quadriceps contraction (Carter et al., 2017). Rotating axis breakage belongs to structural prosthesis failure, but its injury mechanism and improvement methods have been rarely described in the medical literature.

The gastrocnemius is a biarticular muscle with multiple functions for the knee and ankle (Winter, 1980). Heads of the gastrocnemius originate from the medial and lateral femoral condyles, travel downward to form the Achilles tendon with the soleus, and terminate at the calcaneal tuberosity. As a knee flexor, the gastrocnemius antagonizes the quadriceps in synergy with the hamstrings to maintain knee stability. Previous studies have shown that co-contraction of the gastrocnemius with the quadriceps can effectively increase joint compression and reduce anterior tibial translation (Durselen et al., 1995; Sherbondy et al., 2003; Ali et al., 2014; Morgan et al., 2014). Therefore, we hypothesized that it would be feasible to reconstruct the gastrocnemius anatomically to relieve rotating axis stress concentration in distal femoral tumor surgery. In this research, a finite element (FE) model of the lower limb, including a rotating hinge knee (RHK) prosthesis and bone, was established to quantify the impact of gastrocnemius reconstruction on the rotating axis and other prosthesis components during gait.

## Materials and methods

**FIGURE 2:**
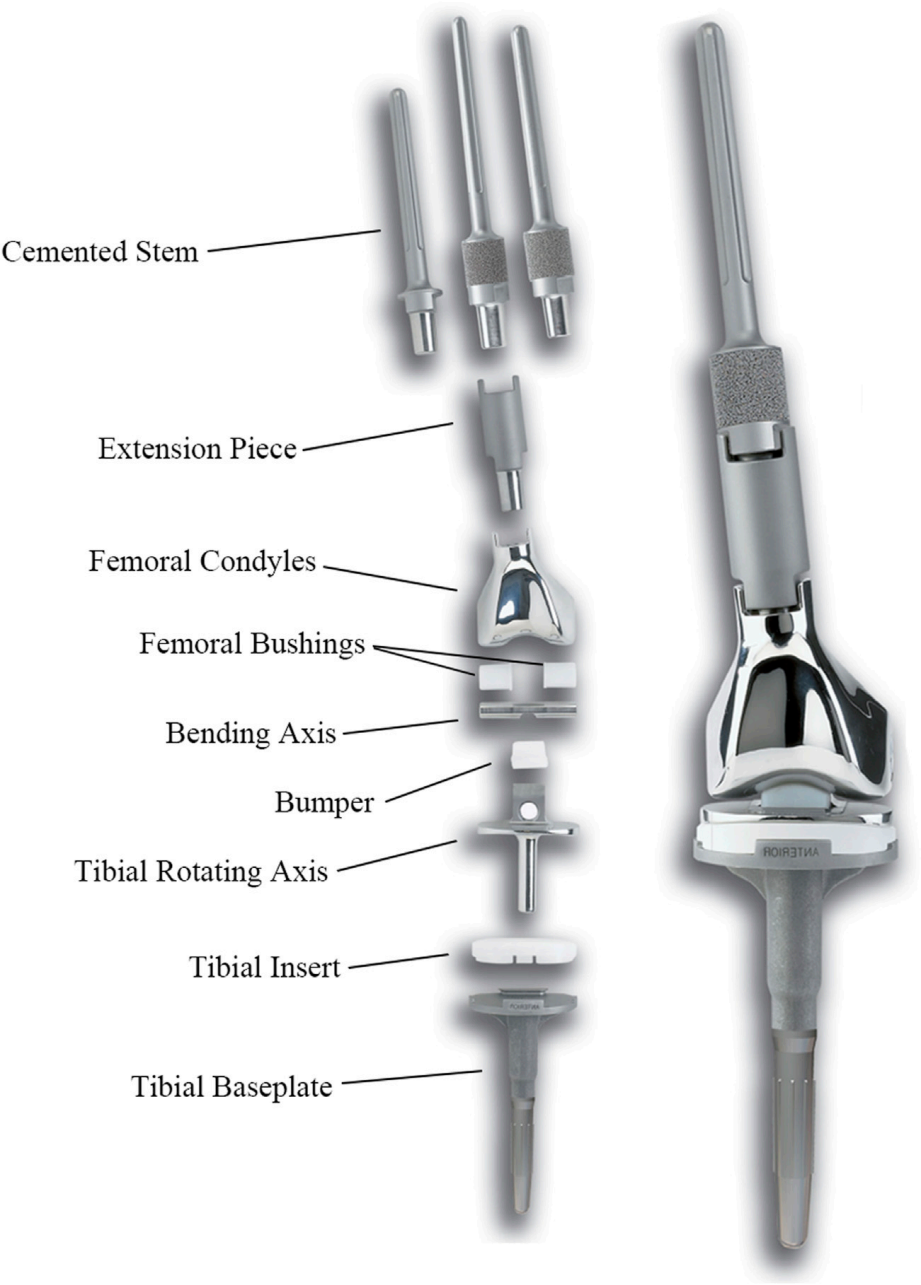
RHK prosthesis used in this study.

The femoral tumor RHK prosthesis was obtained from Beijing Lidakang Company and comprised several components, including the femoral stem, bending axis, rotating axis, tibial baseplate, and UHMWPE spacers (Figure 2). This semi-restrictive prosthesis provides two movable surfaces of flexion/extension and internal/external rotation. The bending axis aligns with the femoral internal and external condylar lines, while the rotating axis is on the line through the middle axis of the tibia. The UHMWPE spacers isolate the intermetallic friction area of the prosthesis, thereby effectively diminishing rotational stress between the components. The clinical outcomes of this prosthesis have been observed to be notably satisfactory, underscoring its efficacy in clinical practice.

### Finite element model construction

This research included a healthy male volunteer (height = 180 cm, weight = 80 kg) after rigorous evaluation and screening parameters, which excluded other knee-related conditions, such as knee deformity and trauma. Signed informed consent was acquired from the volunteer before the experimental program.

Subsequently, the volunteer’s knee joints were carefully scanned through a spiral CT machine in a straightened position, covering the entire length of both lower limbs. For this experiment, the right knee joint data was utilized, and the medical modeling software Mimics (Materialise, Leuven, Belgium) was used to extract the detailed geometric characteristics of the femur and tibia. Furthermore, the data was further refined and smoothed via the engineering software Geomagic (Raindrop Company, United States), which provided comprehensive and accurate solid geometry models of the femur and tibia for later analyses.

**FIGURE 3:**
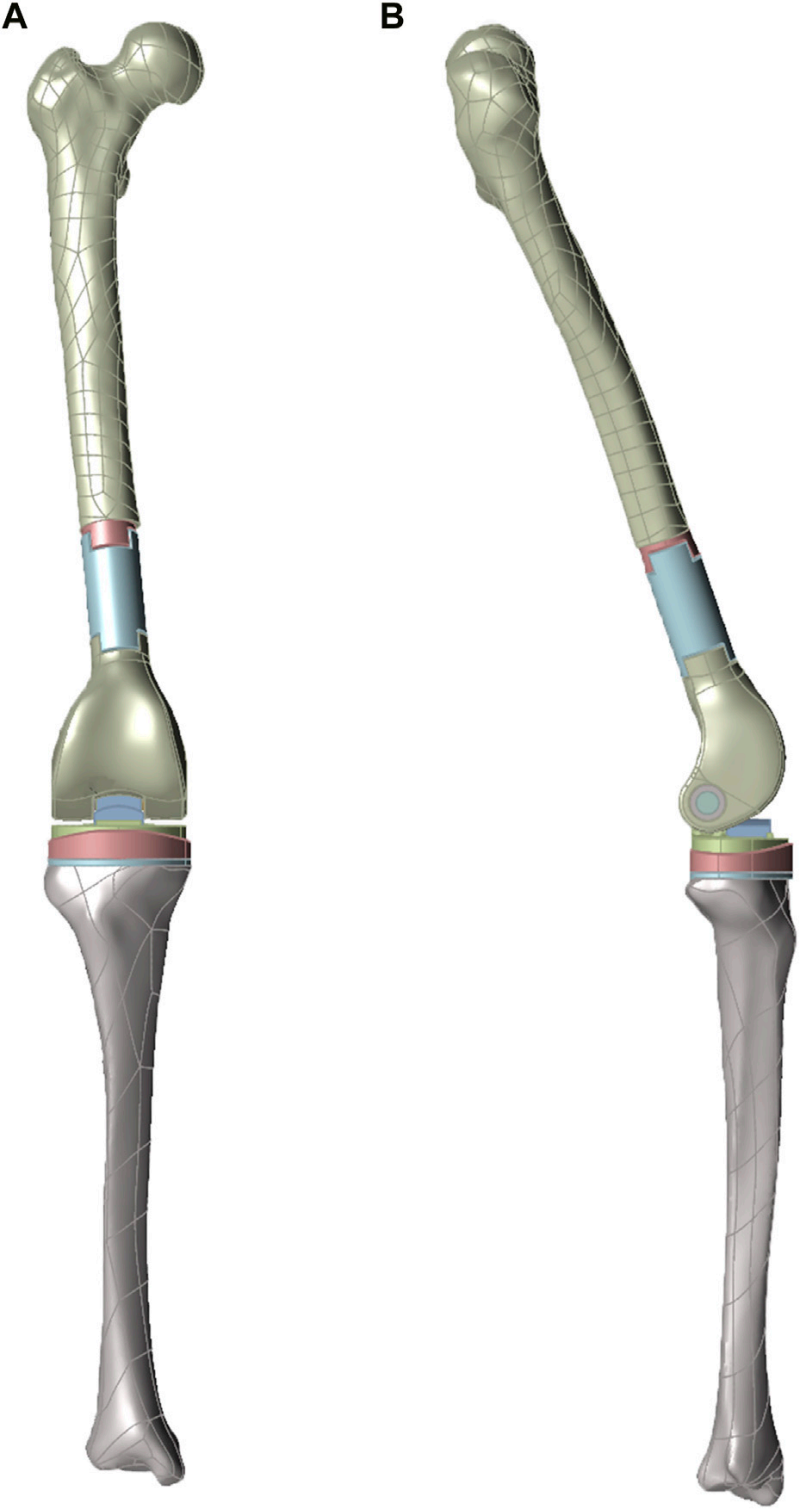
Front **(A)** and side **(B)** view of three-dimensional finite element model.

Following the principle of distal femur tumor resection arthroplasty, the femur segment was removed from a plane about 3–5 cm away from the tumor boundary to prevent tumor recurrence, and the tibia osteotomy was perpendicular to its mechanical axis. Within the model, the midpoint of the knee and ankle joints were marked, and the line connecting the two points was identified as the tibial mechanical axis. For assembling the tumor knee prosthesis, the force line of the lower end of the prosthesis was parallel to the tibial mechanical axis. The acquired 3D model was then imported into ABAQUS 6.14 (Simulia, Providence, RI, United States) for adaptive meshing, and the geometry was divided with a target cell size of 2 mm. Lastly, the mesh file was subjected to static simulation (Figure 3).

### Material properties

The femoral condyles, tibial plateau, bending, and rotating axis of the prosthesis were made of CoCrMo, while the femoral end prosthesis stem and extension were made of TC4 alloy. Table 1 lists the materials used, assigned based on the previous literature (Zhang et al., 2020). The normal and tangential behaviors were set as hard and punishing contacts, respectively. The friction coefficient between CoCrMo and UHMWPE was set at 0.04 during the simulation (Yang and Lin, 2001).

**TABLE 1:** Properties of different materials for knee prosthesis components.

Material	Elastic modulus (MPa)	Poisson’s ratio	Yield strength (MPa)
CoCrMo	220000	0.30	450
TC4	113800	0.34	830
UHMWPE	820	0.46	19
Bone	14000	0.36	215

### Boundary conditions and loading

**FIGURE 4:**
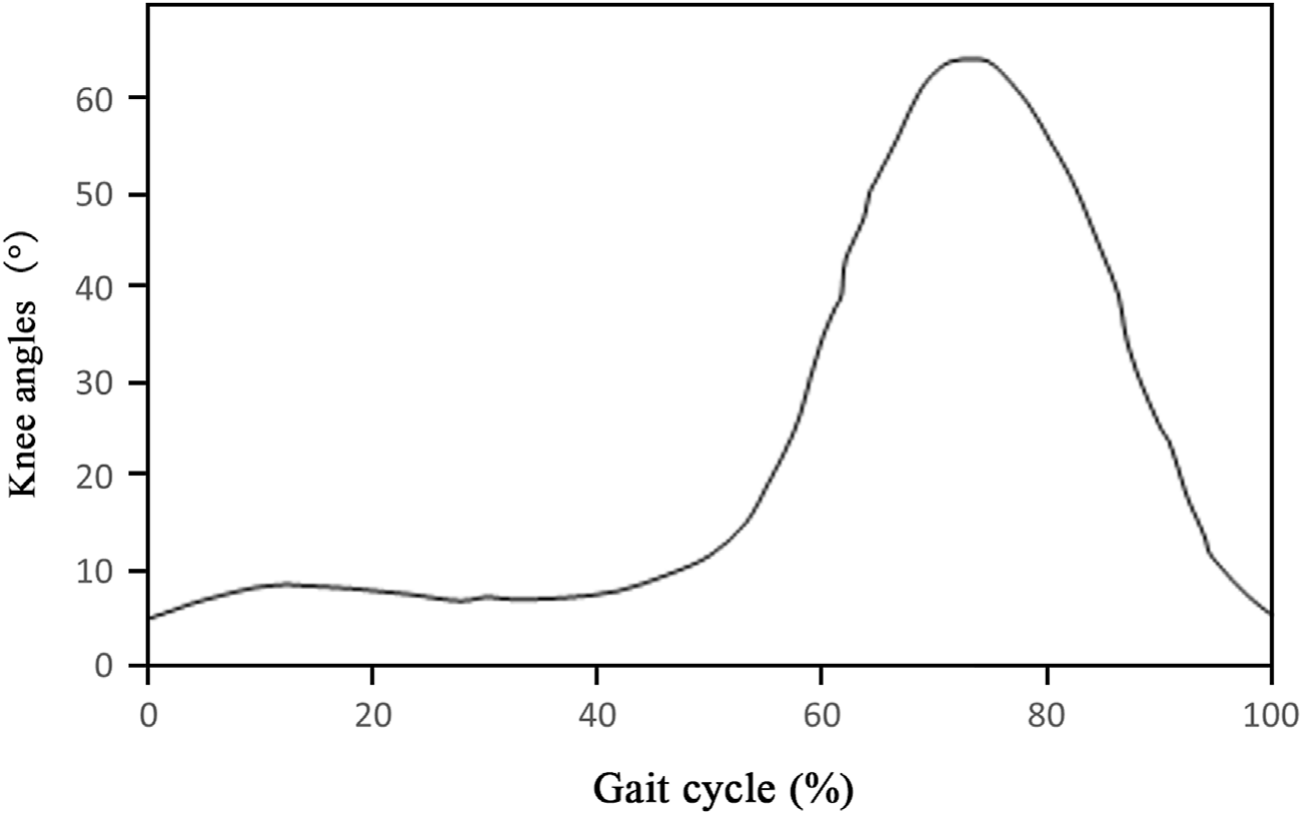
Angle-gait curves after tumor knee arthroplasty.

In order to accurately simulate the mechanical behavior characteristics of the knee joint during gait, the primary knee flexor and extensor muscles were considered in the model, including the quadriceps femoris, biceps femoris, semitendinosus, semimembranosus, and gastrocnemius. The knee motion angle *versus* gait curve after oncologic knee prosthesis replacement was evaluated according to previous literature (Figure 4), which showed that the maximal flexion reached approximately 60° (Okita et al., 2013). In this study, six angles were selected to simulate knee gait after knee prosthesis replacement. Meanwhile, the exact positions of the aforementioned muscles and the muscle force values at each angle were determined based on the anatomical atlas and relevant studies (Table 2) (White et al., 1989; Trinler et al., 2019). The peak Von Mises stress of the prosthesis and peripheral bone tissues was calculated under the loading conditions.

**TABLE 2:** Muscle forces around the knee joint at each angle.

Muscle	Muscle force magnitude at each angle of the knee (N)					
	10°	20°	30°	40°	50°	60°
Rectus femoris	2.10	3.40	3.98	9.87	18.92	29.19
Vastus lateralis	0.86	1.50	1.46	3.15	5.97	11.51
Vastus medialis	0.44	0.44	0.18	0.55	2.45	6.75
Vastus intermedius	2.07	2.07	3.06	4.84	5.91	10.47
Biceps femoris LH	123.52	90.88	50.49	20.01	6.83	7.07
Biceps femoris SH	100.33	80.22	62.99	43.02	29.16	20.06
Semitendinosus	41.32	28.01	17.56	7.42	2.91	3.20
Semimembranosus	233.55	169.23	113.09	43.50	15.73	7.21
Gastrocnemius lateralis	16.50	8.35	4.58	0.75	1.18	1.01
Gastrocnemius medialis	124.28	79.40	47.96	23.79	12.07	9.19

## Results

### Stress distribution of rotating axis

**FIGURE 5:**
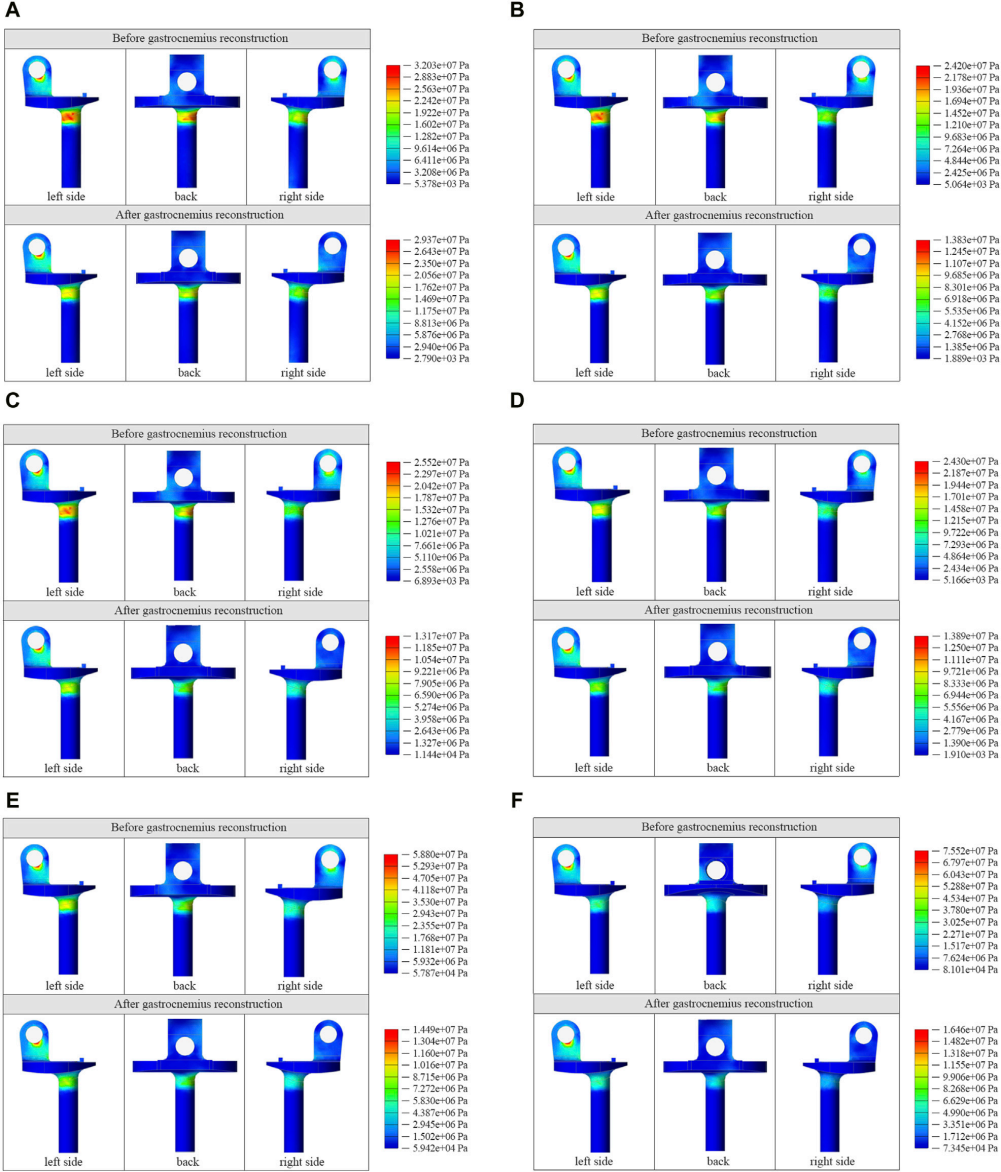
Cloud view of stress distribution in the rotating axis at 60° **(A)**, 50° **(B)**, 40° **(C)**, 30° **(D)**, 20° **(E)**, and 10° **(F)** before and after gastrocnemius reconstruction.

**FIGURE 6:**
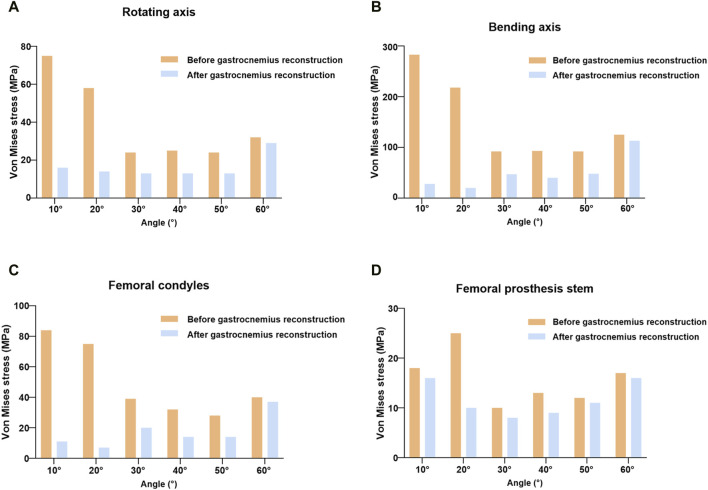
Comparison of peak stress in the rotating axis **(A)**, bending axis **(B)**, femoral condyles **(C)**, and femoral prosthesis stem **(D)** before and after gastrocnemius reconstruction.

The FE model allowed the knee to be stretched from 60° to 10° at 10° intervals, and the stress distribution of the rotating axis before and after gastrocnemius reconstruction was quantified (Figures 5A–F). Before reconstruction, the peak stress was mainly localized in the bushing contact and tibial spacer-wrapped areas, consistent with the actual area of the rotating axis breakage in clinical cases. However, after reconstructing the gastrocnemius, stress was significantly reduced within the tibial spacer-wrapped region. Of the six angles, the unreconstructed group indicated the highest stress at 10°, reaching a peak of 75.52 MPa. Conversely, in the reconstructed group, the maximum stress occurred at 60°, with a peak value of 29.37 MPa. Comparison between the two sets of stress demonstrated that gastrocnemius restoration reduced the peak stress of the rotating axis at all angles, with the most notable reduction at 10°, from 75.52 MPa to 16.46 MPa (Figure 6A).

### Stress distribution in other prosthetic components

Fracture rates of 5%–7% have been observed in oncoplastic knee prostheses, with the prosthesis stem and femoral condyles being common fracture sites (Morgan et al., 2006; Pala et al., 2016). We extracted and compared the peak stress in the bending axis, femoral condyles, and femoral prosthesis stem at different angles. As shown in Figures 6B–D, the trends of stress change in the bending axis and femoral condyles were similar to that observed in the rotating axis. The inclusion of gastrocnemius loading significantly reduced the peak stress levels during knee gait, with the most pronounced effect at 10°, where the bending axis and peak femoral condylar stress decreased from 283.8 to 28.33 Mpa and 84.26 to 11.95 Mpa, respectively. For the prosthesis stem, the maximum peak stress before reconstruction occurred at 20° with 25.21 Mpa and decreased to 10.9 Mpa after gastrocnemius reconstruction. The peak stress of the stem decreased to varying degrees at the other five angles.

## Discussion

This study compares the mechanical environment of the RHK prosthesis before and after rebuilding gastrocnemius by the FE method. To the authors’ knowledge, this is the first to investigate gastrocnemius reconstruction in femoral tumor knee arthroplasty.

In surgical management of malignant bone tumors, it is necessary to remove the involved muscles and soft tissues surrounding the diseased bone to ensure complete resection (DiCaprio and Friedlaender, 2003). Previous studies are mainly focused on the functional restoration of knee extensors and the provision of soft tissue cover (Hobusch et al., 2016; Zan et al., 2023). There is a consensus that robust extensors are critical for post-reconstruction function. On the contrary, rebuilding the flexor muscles, including the gastrocnemius, is usually neglected in distal femoral surgery.

Compared to normal knee arthroplasty, tumor knee arthroplasty presents unique challenges. The incidence of complications related to implant failure ranges from 21.6% to 61% (Bus et al., 2017; Zhang et al., 2018; Christ et al., 2023). Of these, prosthetic breakage is an important cause of revision, which could be attributed to the material used and high mechanical stress. We have noticed some cases of rotating axis breakage. The mechanism of injury may be due to excessive contraction of the quadriceps muscle during knee extension, causing the tibia to move ahead and driving the rotating axis forward. This can further engender localized stress concentrations, heightening the susceptibility to regional structural failure.

Several studies have shown that increased gastrocnemius activation contributes to decreased anterior tibial translation and anterior cruciate ligament (ACL) loading (Ali et al., 2014; Morgan et al., 2014). In musculoskeletal modeling of single-leg landings, the researchers discovered that with co-activation of the quadriceps, enhanced gastrocnemius forces led to stronger joint compression. As a result, increased friction between the knee joints caused by joint compression limited anterior tibial translation, reducing ACL strain (Morgan et al., 2014). Furthermore, another simulation study showed that elevated gastrocnemius activation decreased the peak force of anterior tibial translation, hence lowering the risk of non-contact ACL injury (Ali et al., 2014). Indirect studies have also evaluated the relationship between the gastrocnemius and ACL by detecting muscle activation in ACL-deficient patients. Lass et al. reported that gastrocnemius activity increased in ACL-deficient individuals, indirectly indicating that the gastrocnemius acts as an ACL synergist (Lass et al., 1991). Nevertheless, some investigations have demonstrated that the gastrocnemius muscle can function as an ACL antagonist (Elias et al., 2003; Adouni et al., 2016). When the gastrocnemius muscle contracts and expands in size, it pushes the tibia forward. However, at sufficient gastrocnemius stimulation level, the joint compression is expected to be enough to counter the effects of muscle expansion and restrict the anterior tibial movement (Fleming et al., 2001; Teng et al., 2021).

Consistent with the above theories, our findings suggested that gastrocnemius restoration considerably affected the mechanical environment of the prosthesis. Prior to reconstruction, the stress was concentrated in the area surrounded by the spacer in the rotating axis, which corresponded to the clinical fracture location. The regeneration of gastrocnemius greatly reduced the stress concentration in the fracture-prone region. In RHK prostheses, hinge structures such as the axis of rotating and bending replace the role of ACL, limiting the upper tibia’s anterior movement. As a consequence, gastrocnemius rebuilding provides enough joint compression as well as posterior shear force that resist the anterior tibial translation caused by the quadriceps muscle’s anterior shear force, lowering the strain on the rotating axis. Furthermore, the peak stress in other prosthetic components was reduced to varying degrees after reconstruction, which may prolong the effective period of the artificial knee joint.

Soft tissue reconstruction in endoprosthetic replacement can be accomplished in a variety of ways, including (a) direct fixation to the implant with screws or sutures, (b) synthetic materials, such as LARS ligaments and Trevira tube, and (c) biological augmentation with or without synthetic components (Ek et al., 2011). Reconstruction of the gastrocnemius can be performed in the manners described above, increasing the posterior soft tissue coverage and enhancing prosthetic joint stability.

There are some shortcomings in this study that need to be improved in subsequent experiments. Six angles (10°, 20°, 30°, 40°, 50°, and 60°) were selected to statically simulate the gait, and further refinement of gait angles is required for dynamic simulation. In addition, since the quadriceps and flexor muscles are often partially resected during knee tumor resection surgery, the changes in muscle force distribution need further confirmation.

## Conclusion

This is the first study to propose gastrocnemius reconstruction in tumor knee arthroplasty and verify its feasibility by FE analysis. According to the results, anatomical gastrocnemius restoration in distal femoral arthroplasty can considerably relieve stress concentration on the rotating axis, potentially lowering the risk of prosthetic fracture.

## Data Availability

The original contributions presented in the study are included in the article/Supplementary material, further inquiries can be directed to the corresponding authors.
